# Testing for Universal Common Ancestry

**DOI:** 10.1093/sysbio/syu041

**Published:** 2014-08-12

**Authors:** Leonardo de Oliveira Martins, David Posada

**Affiliations:** Department of Biochemistry, Genetics and Immunology, University of Vigo, Vigo 36310, Spain;

A phylogenetic model selection test to quantify the evidence for the Universal Common Ancestry (UCA) of life forms was proposed recently (Theobald 2010a), based on the comparison of the statistical support, using likelihoods, the Akaike Information Criterion (AIC), or Bayes factors, for two different phylogenetic models representing the UCA and the independent origins (IOs) hypotheses (Sober and Steel 2002). In this test, the former is represented by a single phylogeny connecting all sequences, whereas the latter is depicted by several, independent phylogenetic trees (Fig. 1). Importantly, in the original UCA test, the same alignment was used to represent both hypotheses. When applied to a particular data set of 23 universally conserved proteins, the test strongly favored a UCA scenario.

**Figure 1.**
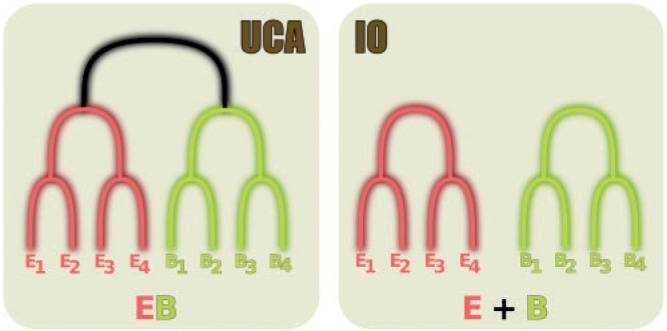
Diagram showing how the UCA and IO hypotheses can be represented by phylogenies, according to Theobald (2010a). Although the UCA assumes that all sequences are connected by one single phylogeny, the IO posits that there is no branch (represented in black) connecting the two domains. It is mathematically equivalent to an infinite length for this branch (see online Supplementary Text S1).

Although there is no question of the common ancestry of the particular set of aligned sequences analyzed, since its publication several criticisms of the test have been raised. Yonezawa and Hasegawa (2010) showed how the UCA test failed to detect that the mitochondrial genes *cytb* and *nd2* are not homologous, to which Theobald replied that when the test is applied to codon or protein models, as originally devised, then the IO hypothesis is correctly preferred (Theobald 2010b). More recently, the same authors extended their analysis and commented on the possible failure of the test for cases of convergence toward similar amino acid composition (Yonezawa and Hasegawa 2012). Koonin and Wolf (2010) simulated alignments lacking phylogenetic structure (site columns came from an independent distribution of amino acid frequencies) and showed that the test would spuriously favor UCA, probably because it was misled by column-wise similarity. In a recent reply, Theobald (2011) included the model used to simulate Koonin and Wolf's data, which was indeed preferred over the UCA model. In his reply, he also suggested that Koonin and Wolf's simulations “were produced by a well-known common ancestry model,” which we believe is incorrect because the IO model described by Theobald (2010a) corresponds mathematically, in the limit, to a tree with at least one infinite branch length (see online Supplementary Text S1; available from http://dx.doi.org/10.5061/dryad.gn376). We also pointed out (Martins and Posada 2012) that the original UCA analysis was affected by selection bias: The query data consisted of sequences already subjected to a similarity search (e.g., BLAST) whose putative column-wise homology status had then been optimized by an alignment algorithm (Brown et al. 2001). In addition, we showed that under the representation of UCA and IO as one versus multiple phylogenies, we can easily distinguish sequences simulated under UCA versus IO by simply observing similarity measures, concluding that similarity should not be used to select which data sets are eligible for the UCA test.

In this point of view, we demonstrate a fundamental drawback of the original UCA test, which is the use of the same alignment to represent both the UCA and IO hypotheses. The UCA test uses the standard phylogenetic likelihood (*L*), which is the probability (*P*) of the “aligned sequences” (*D*) given a phylogenetic hypothesis (*H*; which is UCA or IO), *L* = *P* (*D*|*H*). Phylogenetic studies usually consider alignments as raw data (*D*) and so there is an underlying assumption that all sites from a column are homologous. However, in reality, the unaligned sequences are the true raw data and the fixed alignment should be recognized as a point estimate of the homology relations (Kumar and Filipski 2007; Wong et al. 2008). In any case, in order to make the competing model likelihoods comparable, they have to be based on the same data, which in the original UCA test translates into using a single, fixed global sequence alignment to represent both UCA and IO, even if the global alignment is later split for the calculation of the IO model likelihood. Given the global homology assumption made by multiple alignment programs (Meng et al. 2011; Varon and Wheeler 2012), the possibility that a fixed alignment could bias the test toward UCA has been raised before (Yonezawa and Hasegawa 2010; Theobald 2011), although never demonstrated. In fact, in phylogenetics, alignment algorithms try to optimize the data to conform to a common ancestry hypothesis, and many even use a guide tree, like ClustalW (Thompson et al. 1994) which was the program utilized to align the protein sequences studied by Theobald (2010a). In order to better understand the performance of the UCA test, we performed the simulation study described next, followed by a proposed solution that might alleviate the bias.

## UCA Test Performance under Simulated IO

We simulated sets of sequences evolving under the IO hypothesis, using parameter values estimated from the data in Theobald (2010a), a concatenated data set of 6591 sites of four eukaryotic (*E*) and four bacterial (*B*) sequences. We used INDELible (Fletcher and Yang 2009) to simulate protein evolution (without indels) independently along the *E* and *B* trees under the best-fit amino-acid replacement models in Theobald (2010a) (rtREV + GF), forming two sets of four sequences (quartets) per simulated data set. Both quartets were then grouped together into a single data set composed of eight sequences.

**Figure 2.**
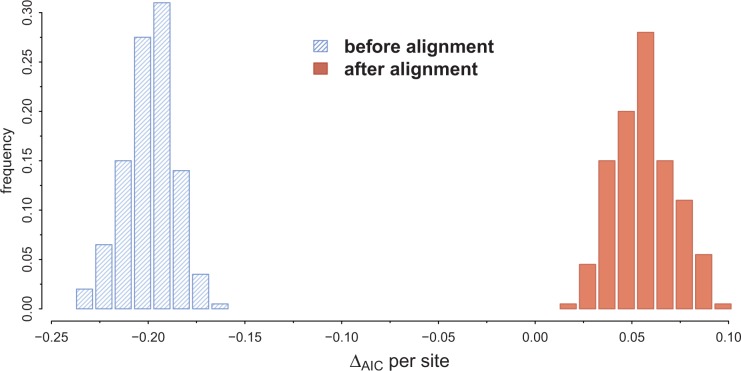
Data set simulations under IO before and after optimizing the alignment, where positive values for ΔAIC suggest a UCA. It shows ΔAIC per site for data sets simulated under the best model and parameters according to the original study (rtREV + GF). The simulated data sets have 6591 sites before optimizing the alignment, and for each parameter set we simulated 200 replicates. All replicates favor IO before aligning the sequences, but then spuriously favor UCA after the alignment step.

Next, we performed the UCA test for the simulated data sets. We aligned all sequences with MUSCLE (Edgar 2004) and estimated the AIC scores for the UCA and IO models as described by Theobald (2010a). These alignments were not subjected to further processing such as removal of gapped columns or regions of low quality, and presented between 7% and 11% of gaps. In Figure 2, we show the results for 200 simulated replicates, where ΔAIC = AIC(*B*) + AIC(*E*) − AIC(*BE*), such that positive values for ΔAIC favor UCA. Clearly, we can see that the UCA hypothesis is incorrectly preferred by a large margin in all simulated data sets.

To investigate whether this bias was caused by the alignment, we implemented the UCA test without the alignment step. As explained above, in the original UCA test, the likelihoods were calculated upon the aligned sequences, so an alignment is the minimum input requirement. As expected, if the alignment operation is not performed (though indels were not simulated so total sequence length was conserved) the test “correctly” favors IO (Fig. 2). Obviously, nobody would (or should) carry out in practice such a phylogenetic test, without aligning the sequences, but this experiment served here to demonstrate that the fixed alignment of the UCA test biases the outcome toward UCA. For the conditions described in Theobald (2010a) and replicated here, the UCA test has a false-positive rate of 100% in our simulations. Our simulations showed that even if one aligns the *E* and *B* subsets independently under the IO hypothesis on the one hand and the *B* + *E* sequences under UCA on the other hand, the AIC (or AICc or BIC) values would still favor UCA (data not shown, scripts available as online Supplementary Material), although we reprove this procedure because the likelihoods compared do not correspond to the same data. This predilection of the test for UCA is due to the fact that the alignment optimization allows for the *B* + *E* sequences to have a much better AIC than their unaligned counterparts, at the cost of adding less than 11% of indels.

Moreover, our reanalysis of previously published data sets purportedly showing the original UCA test favoring IO (Theobald 2010a, 2011) indicates that under proper conditions, the UCA hypothesis is in fact spuriously preferred (Section S2 of the online Supplementary Material). Not surprisingly, given its bias toward UCA, the test always correctly favored the UCA hypothesis for alignments simulated under common ancestry.

We did find other scenarios where the UCA test “correctly” favored IO (results not shown) for the wrong reason, like simulating each life domain under a different amino acid replacement model—which suggests that, in this case, the UCA test is in fact identifying the misspecification of the amino acid replacement model. This implies that whenever the UCA test favors IO, we should further analyze the data before making a decision, since it may not distinguish IO from certain amino acid replacement heterogeneities—an issue already highlighted in Theobald (2010a).

## Reducing the False-Positive Rate of the UCA Test

If we want to reduce the bias toward UCA induced by the alignment step, we should work with the unaligned sequences as our primary data, in order to obtain likelihood values associated to the raw sequences. One way of doing this is estimating the alignment and the phylogeny at the same time (Fleissner et al. 2005; Lunter et al. 2005; Redelings and Suchard 2005, 2007; Novák et al. 2008). Under this framework, the data (*D*) are the (unaligned) sequences, whereas the alignment is one of the parameters of the model, to be treated as an unknown random variable. This type of model is implemented, for example, in the program BAli-Phy (Redelings and Suchard 2005) that not only accounts for substitutions but also explicitly models indels. Therefore, the likelihood values are very different from those obtained by standard phylogenetic models. In order to evaluate the performance of this approach, we simulated protein sequences of 500 amino acids under IO exactly as described before, but this time conducting the test with BAli-Phy instead of MUSCLE + ProtTest + Phyml (under BAli-Phy, the alignment optimization program is redundant). We used BAli-Phy to jointly estimate the posterior distribution of alignments, branch lengths, and of the shape parameter of the gamma distribution for rate variation among sites assuming the LG + *G* (Le and Gascuel 2008) model under a fixed tree topology with variable branch lengths. For each replicate, we ran the software three times: Once for each domain (*E* and *B*) independently (the product of these two analyses gives us the likelihood for the IO model), and once for the eight-sequence *E* + *B* data set (which gives us the likelihood for the UCA model). Although BAli-Phy can also sample from the space of phylogenies, we fixed the topologies at their true values (the ones used in the simulation) and allowed only the branch lengths to vary in the interests of straightforward computation.

**Figure 3.**
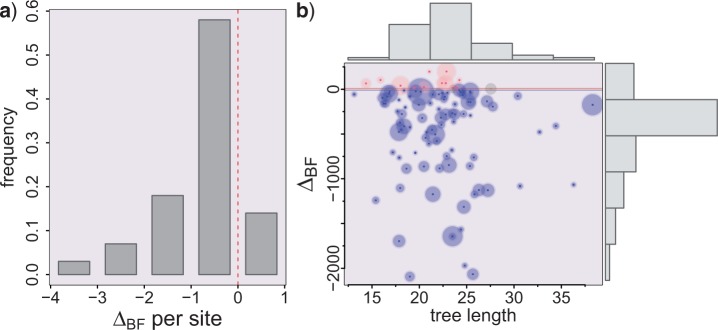
Bali-Phy results for IO simulated data sets. a) Histogram of the log Bayes factor values per site as calculated by ΔBF divided by the posterior median of the alignment length for the BE data set. b) Unscaled ΔBF against posterior median estimate of tree length under the UCA hypothesis, for 100 replicates. The circle diameter represents the posterior median alignment length for the BE data set, going from 506 to 868 sites. The 12 data sets shown at the top wrongly support UCA, whereas the gray circles near zero are two inconclusive simulations, assuming that more than 10 BF units between the hypotheses corresponds to strong evidence. The correctly identified IO data sets are shown at the bottom part, below -10 BF units.

We used the marginal likelihoods calculated as the harmonic mean of the sample likelihoods (Kass and Raftery 1995), in order to estimate the Bayes factor between the UCA and IO hypotheses. Notice that for each replicate, we will have an alignment distribution for *B* only, then one for *E* only, and finally one for *B* + *E*, together with their respective marginal likelihoods *P*(*B*), *P*(*E*), and *P*(*B* + *E*). Therefore, we have ΔBF = log[Prob(*D*/UCA)] − log[Prob(*D*/IO)] = log[*P*(*B* + *E*)] − log[*P*(*B*)] − log[*P*(*E*)], such that positive values support UCA. In Figure 3, we show the results from 100 replicates, where we can see that 86% of the simulations were correctly identified as supporting IO, 12% favored UCA, and two simulations were inconclusive. Figure 3a shows the histogram with ΔBF values normalized per site—that is, divided by the posterior median alignment length—whereas Figure 3b plots the raw ΔBF values against the posterior median total tree length. Note that there is no apparent correlation between tree length and support for UCA. Here, we must note that these Bayes factors should not be taken at face value: The harmonic mean estimator (HME) is numerically unstable and tends to favor more complex models, and although better estimators exist, they are not implemented yet in most Bayesian phylogenetic software (Lartillot and Philippe 2006; Xie et al. 2011). The HME also tends to overestimate the marginal likelihood, which will favor IO more easily (Lartillot and Philippe 2006). In any case, we believe that these results clearly suggest that considering alignment and phylogeny coestimation should reduce to a large extent the bias toward UCA evidenced by the original UCA test.

## Discussion

We have shown that the UCA test described in Theobald (2010a) is unable to detect the IOs of two sets of unrelated sequences. Although our simulations are not exhaustive—we did not explore many possible combinations of trees, branch lengths, sequence sizes, and evolutionary models for instance—they show that there are many cases not unlike real data sets where the UCA test fails. Our general impression is that the original UCA test would not reject a common origin for any but obviously unrelated set of sequences. Certainly, one can argue that for a specific, particular data set, the UCA test has worked. But the high “quality” of the original data set should not be used to justify the correctness of the method. We have previously noted (Martins and Posada 2012) that selecting the sequences based on similarity can make the alignment bias disappear due to the lower number of introduced indels, but then this selection procedure clearly introduces its own bias.

Theobald (2011) offered a few suggestions for situations when we are not very confident about the alignment. The first was to use structural alignments, which might be a promising approach in the future but depends on the ability of structurally aligning simulated or empirical independent sequences of arbitrary similarity. The second was to account for “alignment bias and uncertainty,” which according to our simulations is in fact a prerequisite if the UCA test is to be applied as devised. Moreover, we believe that any formal attempt to quantify the UCA hypothesis must take into account the selection and alignment of sequences into the test. The third suggestion was a permutation procedure whereby sites for certain sequences are shuffled followed by recalculation of the AICs after realignment. This would tell us by how much the original data depart from data sets whose phylogenetic structure has been partially removed. However, using AIC to compare different data sets is not a valid approach. Therefore, AIC values between distinct alignments cannot be interpreted in probabilistic terms. Still, this procedure can lead to a permutation test (similar to the permutation tail probability tests of Faith and Cranston [1991] and Swofford et al. [1996]), in which a wide collection of test statistics can be used in place or in addition to the ΔAIC.

The full BAli-Phy analysis on each of the 500 sites replicate took more than 1 week on a single thread, even assuming a fixed topology, restricting right now these type of analyses to small data sets. In any case, any data set must be aligned to be amenable to the original UCA test, and here we have demonstrated that by doing so the test will often favor UCA. We want to emphasize again that we are not denying the common ancestry of the data set analyzed in Theobald (2010a). What we and others have been pointing out are shortcomings of the UCA test itself.

## Supplementary Material

Data available from the Dryad Digital Repository: http://dx.doi.org/10.5061/dryad.gn376.

## Funding

This work was supported by the European Research Council [ERC-2007-Stg 203161-PHYGENOM to D.P.].
